# Aristolochic Acid and Hepatocellular Carcinoma: A Critical Review of Genotoxic and Inflammatory Mechanisms

**DOI:** 10.3390/ijms27114746

**Published:** 2026-05-25

**Authors:** Yupeng Wang, Yikun Zhang, Tianqi Ren, Liyong Yuan, Xingchao Geng

**Affiliations:** 1 Institute for Biological Product Control, National Institutes for Food and Drug Control, Beijing 102629, China; wangyp@nifdc.org.cn; 2 National Center for Safety Evaluation of Drugs, National Institutes for Food and Drug Control, Beijing 100176, China; zhangyikun0522@163.com (Y.Z.); 3323092134@stu.cpu.edu.cn (T.R.)

**Keywords:** aristolochic acid, hepatocellular carcinoma, genotoxicity, inflammation, DNA adducts

## Abstract

Aristolochic acid (AA), a naturally occurring compound found in Aristolochia plants, is a well-established nephrotoxin and Group 1 carcinogen. Emerging evidence suggests a potential link between AA exposure and hepatocellular carcinoma (HCC), one of the leading causes of cancer-related mortality worldwide. This review critically evaluates current knowledge on AA’s hepatic metabolism, its formation of persistent DNA adducts, and the induction of inflammatory responses in the liver. Based on preclinical and indirect human evidence, we propose a working hypothesis that AA may contribute to hepatocarcinogenesis through a dual mechanism: genotoxic (primarily via H-ras and p53 mutations resulting from AA-DNA adducts) and non-genotoxic (via chronic inflammation involving IL-6, TNF-α, and NF-κB activation, as well as epithelial–mesenchymal transition). We note, however, that these mechanisms remain to be validated in human cohorts and do not yet establish causality. Recent studies have identified novel mechanisms, including PDK4-mediated mitochondrial dysfunction, ferroptosis inhibition via p53 hijacking, and ARID1A deficiency as a susceptibility factor. A recent meta-analysis quantified a significantly increased risk of liver cancer following AA exposure in epidemiological studies. While direct causal evidence in humans remains limited, the high mutational burden observed in AA-exposed liver tissues warrants caution. Nevertheless, the primary public health priority pertains to the prevention of AA exposure. Further epidemiological and mechanistic studies are urgently needed.

## 1. Introduction

Aristolochic acid (AA) and its derivatives are nitrophenanthrene carboxylic acids isolated from Aristolochia species, including over 400 species worldwide [1]. The primary components are aristolochic acid I (AAI) and aristolochic acid II (AAII), which have historically been used in traditional medicine for conditions such as arthritis, gout, rheumatism, and snake bites [2]. However, AA was later found to cause severe nephrotoxicity and upper urinary tract urothelial carcinoma (UTUC) [3,4]. The International Agency for Research on Cancer (IARC) has classified AA as a Group 1 carcinogen [5]. Hepatocellular carcinoma (HCC) is the third leading cause of cancer-related death globally, with more than half of new cases occurring in China [6]. The high incidence of HCC in China, combined with the widespread historical use of AA-containing herbs, has raised concerns about a potential causal link. A landmark study published in Science Translational Medicine suggested that AA exposure might be associated with liver cancer across Asia [7]. This finding sparked a global debate on whether AA contributes to liver carcinogenesis [3]. Subsequent studies have detected AA-specific mutational signatures in liver cancer tissues across multiple Asian populations [8,9]. A recent meta-analysis further quantified this association, demonstrating a significantly increased risk of liver cancer following AA exposure (OR: 1.146, 95% CI: 1.040–1.262) [10]. Beyond HCC, the AA mutational signature has also been recently identified in hepatic angiosarcoma, expanding the spectrum of AA-associated liver malignancies [11]. However, the causal relationship remains controversial. Some studies have reported that long-term follow-up of patients with AA nephropathy (AAN) did not reveal an increased incidence of HCC [12], while others have proposed a “spatiotemporal heterogeneity” hypothesis—suggesting that AA may exert stronger hepatocarcinogenic effects in juvenile individuals than in adults—to explain the inconsistent findings across different age groups [13]. This review does not claim causality; rather, it synthesizes existing evidence to generate testable hypotheses for future research.

Most existing research has focused on AA-induced nephrotoxicity, with relatively little attention paid to the liver. Moreover, traditional toxicological studies often concluded that the liver is not a target organ for AA because histopathological lesions were not consistently observed [14]. However, emerging evidence from high-throughput sequencing has revealed significant genotoxic effects in the liver even in the absence of visible pathology [9]. This review aims to: (1) summarize current knowledge on AA metabolism and DNA adduct formation in the liver; (2) critically evaluate evidence for AA-induced liver damage, including both genotoxic and non-genotoxic mechanisms; (3) incorporate recent discoveries including ferroptosis, PDK4-mediated mitochondrial dysfunction, and ARID1A deficiency; and (4) propose an updated dual-mechanism framework for understanding AA’s potential role in hepatocarcinogenesis. Finally, we discuss clinical implications and priorities for future research.

## 2. Materials and Methods

Literature search strategy: A comprehensive literature search was performed in PubMed, Scopus, and Web of Science databases. The search was conducted from database inception to March 2026. The following Medical Subject Headings (MeSH) and related entry terms are used to search English literature. Medical Subject Headings include: “Carcinoma, Hepatocellular” and “aristolochic acid I”. Entry Terms include: “Hepatoma”, “Liver Cancer, Adult”, “Adult Liver Cancers”, “Carcinoma, Liver Cell”, “Liver Cell Carcinomas”, “8-methoxy-6-nitrophenanthro(3,4-d)-1,3-dioxole-5-carboxylic acid”, “aristolochic acid”, “Tardolyt”, “sodium aristolochate”, “aristolochic acid I, sodium salt” (note: literature retrieval operators “AND”, “OR” and “NOT” are used to link MeSH with Entry Terms). In addition, we also performed a manual search using the reference list of key articles published in English.

Study selection: A total of 184 records were initially identified: 56 from PubMed, 72 from Scopus, 48 from Web of Science, and 8 from other sources (reference lists of key reviews). After the removal of 47 duplicate records, 137 articles remained.

Two authors (Yupeng Wang and Yikun Zhang) independently screened the titles and abstracts of the retrieved records. Studies were excluded according to the following criteria: (1) articles not written in English (*n* = 12); (2) commentaries, editorial letters, or surveys (*n* = 8); (3) studies not directly relevant to AA and liver carcinogenesis based on title and abstract (*n* = 42). The full texts of the remaining 75 articles were assessed for eligibility. Nine articles were excluded because the full text could not be accessed, and five articles were excluded due to a lack of direct relevance to the research question after full-text review. Final inclusion: A total of 61 articles met the inclusion criteria and were included in this review. Disagreements between the two reviewers were resolved by discussion or consultation with a third author.

## 3. Hepatic Metabolism of AA and Formation of DNA Adducts

### 3.1. Metabolic Activation in the Liver

The toxicity of AA is closely linked to its metabolic processing. In the liver, AAI is primarily activated by NAD(P)H: quinone oxidoreductase 1 (NQO1) and cytochrome P450 (CYP) 1A1/2 through nitroreduction [15,16]. This process generates N-hydroxyaristolactam I, which forms a cyclic N-acylnitrenium ion—the ultimate carcinogenic species [17].

This electrophilic ion can bind to DNA or undergo structural rearrangement to form 7-hydroxyaristolochamide I [18]. NQO1 plays a particularly important role in AAI activation. Studies have shown that AAI binds to NQO1 to form an NQO1-AAI complex, which facilitates reduction and subsequent DNA binding [19].

CYP1A1/2 also contribute significantly to AAI activation in the liver [20]. The nitro group has been identified as the primary toxic moiety of AA, with methoxy and hydroxyl groups further enhancing toxicity [21]. Recent structural biology studies have provided new insights into AA transport. Pomyalov et al. solved the crystal structure of human serum albumin (HSA) in complex with AA at 1.9 Å resolution, revealing the molecular basis of AA distribution in the body [22].

In summary, the liver possesses the full enzymatic machinery (NQO1, CYP1A1/2) to metabolically activate AA into a highly reactive nitrenium ion capable of forming DNA adducts.

### 3.2. AA-DNA Adducts

AA forms persistent DNA adducts through covalent binding to purine bases. The major adducts include 7-(deoxyadenosin-N^6^-yl) aristolactam I (dA-AAI), 7-(deoxyguanosin-N^2^-yl) aristolactam I (dG-AAI), and the corresponding AAII-derived adducts (dA-AAII, dG-AAII) [23]. dA-AAI is the most abundant and persistent adduct in target tissues, with a half-life of months to years [24].

dA-AAI is a mutagenic lesion that preferentially induces A: T → T: A transversions in vitro [25]. This specific mutational signature has been detected in the p53 gene of patients with AA nephropathy [26]. Importantly, AA-DNA adducts are difficult to repair, and their long-term persistence leads to accumulated mutations that may initiate carcinogenesis [27].

Notably, even in the absence of histopathological liver lesions, AA-DNA adducts have been detected in rodent liver tissues following AA administration [9]. In a study by Lu et al., AA-treated mice showed significant levels of dA-AAI in the liver, accompanied by hundreds of gene mutations despite normal liver histology [8].

Recent mechanistic studies have uncovered unprecedented free radical mechanisms for AA-DNA adduct formation. Li et al. demonstrated that AA metabolites generate free radicals that directly attack DNA, providing a new perspective on AA genotoxicity [28].

### 3.3. Novel In Vitro Models for Studying AA Genotoxicity

Recent advances in cell culture technology have provided more physiologically relevant models for studying AA hepatotoxicity. Caipa Garcia et al. used three-dimensional organoid cultures derived from human stomach, liver, kidney, and colon to study AAI metabolic activation. They found that organoids from target tissues (kidney) exhibited higher levels of DNA adduct formation compared to non-target tissues at equivalent cytotoxicity levels [29].

More importantly, Hu et al. used functional human-induced hepatocyte-like cells (hiHep cells) to evaluate AAI genotoxicity. This model closely mimics primary human hepatocytes in gene expression and function. They found that AAI (0.7–2.5 µM) induced up to 105 adducts per 10^8^ nucleotides in hiHep cells, indicating efficient metabolic activation.

AAI also increased micronucleus frequency in a concentration-dependent manner and significantly elevated 8-hydroxy-2′-deoxyguanosine and reactive oxygen species levels, suggesting oxidative DNA damage. Importantly, ascorbic acid treatment reduced AAI-induced oxidative DNA damage and DNA adduct formation, providing direct cellular evidence for free radical intermediates in AAI metabolic activation [30].

In summary, AA forms persistent, mutagenic DNA adducts in the liver, with dA-AAI being the most stable. Novel human-derived cell models (hiHep cells, organoids) have confirmed significant genotoxicity of AA in human-relevant systems, with oxidative stress emerging as a key mechanism.

## 4. Evidence for AA-Induced Liver Damage and Mutagenesis

### 4.1. Genomic Mutations in the Liver

High-throughput sequencing has revolutionized our understanding of AA’s effects on the liver. In a whole-genome and exome analysis of AA-associated UTUC patients, Han and colleagues found that AA-exposed tissues exhibited a strikingly high somatic mutation rate of 150 mutations per megabase in the liver—far exceeding that of smoking-associated lung cancer (8 mutations/Mb) and comparable to UV-associated melanoma (111 mutations/Mb) [8].

In animal studies, Mei et al. demonstrated that AA induces a dose-dependent increase in liver gene mutation frequency in rats [31]. Poon et al. identified AA-specific mutational signatures in liver cancer tissues from patients with known AA exposure, providing direct evidence linking AA to genomic alterations in the human liver [9].

A particularly revealing study by Chen et al. compared gene expression profiles in the kidneys and livers of Big Blue transgenic rats treated with AA for 12 weeks [32]. While the kidney showed 372 significantly mutated genes involved in carcinogenesis, the liver showed 111 significantly mutated genes, primarily regulating lipid metabolism. This tissue-specific difference may explain why the kidney is more visibly affected than the liver in traditional toxicology studies, despite both organs accumulating DNA damage.

Recent large-scale genomic studies have provided additional validation. Chen et al. performed deep whole-genome analysis of 494 hepatocellular carcinomas, providing a comprehensive landscape for validating AA mutational signature frequencies [33]. Fang et al. used integrated transcriptomic and metabolomic approaches to compare hepatotoxicity between neonatal and adult mice, revealing age-dependent differences in AA susceptibility [34].

### 4.2. Discordance Between Histopathology and Genotoxicity

An important theme emerging from the literature is the discordance between histopathological findings and molecular evidence of genotoxicity. Several studies reported no observable liver lesions after AA treatment [14,35], leading to the conclusion that the liver is not a target organ. However, subsequent molecular analyses revealed substantial DNA adduct formation and gene mutations in the same tissues [8,9].

For example, female Hupki mice treated with AA for 3 weeks showed no pathological changes in the liver, yet AA-DNA adducts were significantly elevated, and 650 gene loci were mutated [9]. Network analysis revealed that pathways involved in apoptosis, NF-κB signaling, and oxidative phosphorylation (e.g., COX7b, ATP5h) were altered.

Similarly, Ke et al. found that continuous AA administration for 10 days caused acute liver inflammation and precancerous lesions in beagle dogs, with overexpression of c-Myc and lin28b [36]. Importantly, Yang et al. reported that liver damage occurred earlier than kidney damage in beagle dogs following AA exposure [37].

**Figure 1:**
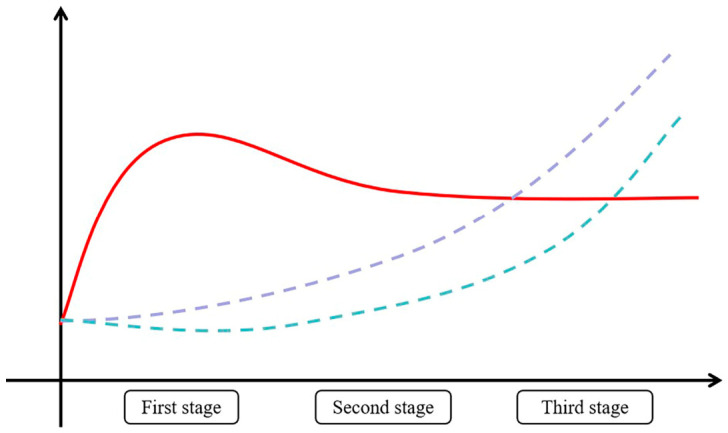
Temporal discordance between AA-DNA adduct formation, mutation accumulation, and histopathological changes in the liver following AA exposure. Following AA exposure, AA-DNA adducts (primarily dA-AAI) form rapidly and persist at elevated levels for extended periods (red solid line). Gene mutations accumulate progressively over time in a dose-dependent manner (purple dashed line). In contrast, histopathological lesions (green dashed line) become detectable only after prolonged exposure or at later time points, despite ongoing genomic damage. This temporal discordance explains why traditional toxicology studies, which rely primarily on histopathology, may underestimate the liver’s susceptibility to AA-induced genotoxicity. Data synthesized from.

Ragi et al. developed an untargeted DNA adductomics approach to screen DNA damage in rat kidney and liver simultaneously, enabling comprehensive assessment of tissue-specific adduct profiles [38]. In summary, the absence of histopathological liver lesions does not rule out AA-induced genotoxicity. Molecular evidence consistently demonstrates that AA causes DNA adduct formation, gene mutations, and pathway alterations in the liver, suggesting that the liver is more vulnerable than previously recognized. The temporal discordance illustrated in Figure 1 is a key observation from the literature [8,9,24]. AA-DNA adducts form rapidly after exposure and persist for extended periods (red line), whereas gene mutations accumulate progressively over time (purple dashed line). In contrast, histopathological lesions (green dashed line) become detectable only after prolonged exposure or at later time points. This temporal discordance has important implications: traditional toxicology studies that rely primarily on histopathology as an endpoint may substantially underestimate the liver’s susceptibility to AA-induced genotoxicity. It also suggests that the absence of visible liver lesions does not rule out ongoing genomic damage that could contribute to long-term carcinogenic risk.

## 5. Dual Mechanisms: Genotoxicity and Inflammation in HCC Development

Before presenting the mechanistic evidence, it is important to clarify the hierarchy of evidence in AA-related carcinogenesis research. Evidence can be categorized into four levels: (1) in vitro mechanistic studies (e.g., cell lines, biochemical assays), (2) animal models (rodents, dogs), (3) human mutational signatures and biomarker studies, and (4) epidemiological association and causation. Most available evidence for AA in HCC falls into levels 1–3. Direct evidence for causation (level 4)—such as prospective cohort studies with well-characterized AA exposure and adequate control for confounders—remains limited. In particular, while AA-DNA adducts and AA-specific mutational signatures are strong indicators of AA exposure and biological plausibility, they do not by themselves prove that AA initiated the specific tumor in a given patient. Causality requires additional evidence from prospective epidemiology and functional validation in human tissues. The following sections describe mechanistic findings with this hierarchy in mind; findings are presented as hypotheses that require further validation in human populations.

**Figure 2:**
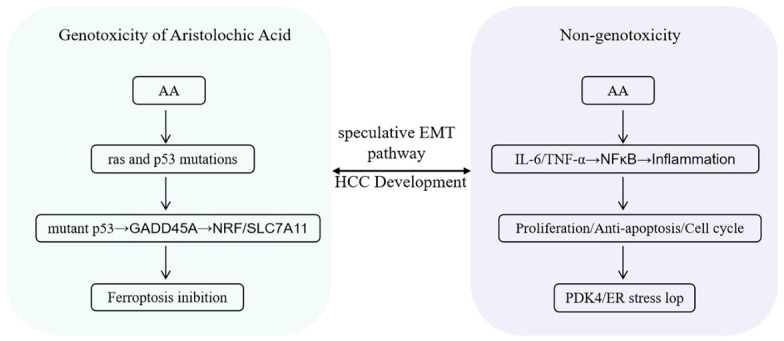
Dual mechanisms of aristolochic acid (AA) in hepatocarcinogenesis. The left panel illustrates the genotoxic pathway: AA is metabolically activated by NQO1 and CYP450 enzymes to form a cyclic nitrenium ion, which binds to DNA to generate persistent dA-AAI adducts. These adducts induce mutations in H-ras and p53, leading to uncontrolled cell proliferation. A novel link between p53 mutation and ferroptosis inhibition is also depicted. The right panel illustrates the non-genotoxic/inflammatory pathway: AA triggers the release of pro-inflammatory cytokines (IL-6, TNF-α) from hepatocytes and immune cells, activating NF-κB signaling. NF-κB promotes cell proliferation, anti-apoptosis, and epithelial–mesenchymal transition (EMT). Both pathways converge to promote hepatocellular carcinoma (HCC) development, with synergistic effects between genotoxicity and inflammation.

HCC development is a protracted, multi-step process involving the accumulation of genetic mutations and chronic inflammation [39]. AA appears to affect both of these key drivers. We propose a dual-mechanism framework in which AA may contribute to hepatocarcinogenesis through: (1) direct genotoxic effects (gene mutations) and (2) non-genotoxic effects (inflammatory microenvironment changes) (Figure 2).

### 5.1. Genotoxic Mechanisms

#### 5.1.1. Ras and p53 Mutations

AA induces mutations in key cancer-related genes in the liver, including the ras proto-oncogene family and the p53 tumor suppressor gene, which may collectively initiate or promote hepatocarcinogenesis. The ras gene family (H-ras, K-ras, N-ras) plays critical roles in cell proliferation and differentiation, and activating mutations in these genes are common in various cancers. Schmeiser et al. first demonstrated that AAI-induced tumors in the rat forestomach and ear duct were associated with c-Ha-ras activation [25]. Subsequent studies found that AA treatment induced dose-dependent H-ras mutations in rodent livers, with a significant elevation of the H-ras codon 61 CTA mutation [40]. In diethylnitrosamine (DEN)-induced rat HCC models, H-ras activation promotes Ras-GTP complex formation and downstream signaling [41]. Given that AA shares a common metabolic activation pathway involving nitroreduction with DEN, it has been proposed that AA may act through similar mechanisms [42]. Meanwhile, the p53 tumor suppressor gene is mutated in approximately 50% of HCC cases [43]. p53 mutations have been well documented in AA nephropathy and AA-associated upper tract urothelial carcinoma, with the mutational spectrum including characteristic A: T → T: A transversions at specific codons [26]. In the liver, p53 mutations affect the formation of oval cells (hepatic progenitor cells), which play an important role in HCC initiation [44]. Furthermore, p53-mutant cells exhibit greater resistance to apoptosis and radiotherapy compared to wild-type cells, suggesting a survival advantage for the mutated cells [45]. Notably, the mutational signature induced by AA in the liver resembles that of aflatoxin B1—a well-established hepatocarcinogen that also forms DNA adducts and induces p53 codon 249 mutations [46,47].

#### 5.1.2. Ferroptosis Inhibition

Beyond direct mutagenesis, a groundbreaking study has uncovered an additional genotoxic mechanism by which AA promotes liver cancer cell growth: hijacking p53 to inhibit ferroptosis, a newly discovered form of regulated cell death. Mechanistically, AA-induced mutant p53 upregulates GADD45A, which in turn activates the NRF2/SLC7A11 axis. This signaling cascade leads to reduced lipid peroxidation and confers resistance to ferroptosis. This represents a previously unrecognized mechanism through which AA promotes hepatocarcinogenesis [48].

Critical note on human validation: Most evidence for this pathway is derived from HCC cell lines and rodent models. Direct evidence in human HCC tissues is currently lacking and represents a priority for future research. Whether this mechanism operates in human livers with chronic AA exposure remains to be determined.

### 5.2. Non-Genotoxic Mechanisms: Inflammation and Epithelial–Mesenchymal Transition (EMT)

Chronic inflammation is a key driver of HCC, particularly in the context of viral hepatitis and non-alcoholic steatohepatitis [49]. Studies have shown that AA can induce inflammatory responses in the liver through multiple pathways, thereby creating a pro-tumorigenic microenvironment. Regarding inflammatory cytokines, Marin et al. found that AA increased the synthesis of TNF-α, IFN-γ, IL-1β, IL-6, and IL-8 in porcine liver [50], while Ke et al. reported that AA-induced acute liver inflammation in beagle dogs was accompanied by elevated IL-6 levels and the emergence of hepatoblastoma-like cells [36]. As a key pro-inflammatory cytokine, IL-6 activates downstream NF-κB signaling, which promotes hepatocyte proliferation and inhibits cell death while also regulating cell cycle proteins (cyclin D1, D2, D3, c-Myc, CDK2, CDK4, CDK6) and COX-2, thereby creating a favorable microenvironment for tumor growth [51,52]. In addition to cytokine-mediated inflammation, recent research by Wang et al. identified a novel mechanism linking AA exposure to hepatocyte apoptosis, demonstrating that AA upregulates PDK4 expression, which in turn promotes p53-mediated ER stress and mitochondrial dysfunction, accompanied by M1 macrophage activation and subsequent hepatic inflammation, thus creating a vicious cycle of liver injury [53]. Furthermore, epithelial–mesenchymal transition (EMT), a critical process in cancer metastasis, has been documented in AA-associated UTUC and nephropathy [54]. TGF-β, a master regulator of EMT, has been shown to induce EMT in HepG2 cells and promote HCC progression and metastasis in the liver [55,56]. Given that AA also induces TGF-β-related pathways in the kidney, similar mechanisms may operate in the liver, although direct evidence is still needed.

In summary, AA contributes to hepatocarcinogenesis through dual mechanisms: on the genotoxic side, it induces mutations in key cancer-related genes (ras and p53) and inhibits ferroptosis via p53 hijacking; on the non-genotoxic side, it promotes inflammatory cytokine production (IL-6, TNF-α), activates NF-κB signaling, and may induce EMT-related pathways. Together, these mechanisms create a pro-tumorigenic microenvironment that facilitates HCC development. Table 1 provides a comprehensive summary of the key evidence supporting this dual-mechanism framework.

## 6. Genetic Susceptibility: ARID1A Deficiency as a Risk Factor

A major breakthrough in understanding individual susceptibility to AA-induced HCC came from Wang et al., published in Advanced Science, who demonstrated using liver-specific Arid1a-deficient mouse models that ARID1A deficiency dramatically accelerates AA-induced liver tumorigenesis. Mechanistically, ARID1A deficiency exerts a dual effect: it downregulates key nucleotide excision repair (NER) genes, compromising the cell’s ability to repair AA-DNA adducts, while simultaneously upregulating Nqo1 expression, enhancing the metabolic activation of AA; this combination leads to the accumulation of characteristic Ctnnb1 mutations and significant alterations in the liver microenvironment. Notably, ARID1A is mutated in over 20% of human cancers, and these mutations frequently occur in non-cancerous tissues of elderly individuals, suggesting a broad population at risk [57]. From a clinical perspective, ARID1A mutation status may serve as a biomarker for identifying individuals at elevated risk for AA-induced HCC, and patients with known or suspected ARID1A deficiency should be particularly cautious about AA exposure.

## 7. Clinical Implications and Risk Considerations

### 7.1. Evidence Supporting a Potential Association

The evidence reviewed above has several important clinical implications. First, for patients with pre-existing liver disease (e.g., chronic hepatitis B or C, non-alcoholic fatty liver disease, cirrhosis), exposure to AA may further increase their cancer risk, as the combination of AA-induced genotoxicity and the underlying inflammatory milieu of diseased livers may accelerate HCC development. This situation is analogous to the dramatic increase in HCC risk associated with aflatoxin B1 exposure in HBV-endemic regions [58].

Second, even in individuals without known liver disease, long-term or repeated exposure to AA may lead to the persistent accumulation of DNA adducts and mutations, an effect that can last for decades [24]. Although these “silent” mutations do not cause immediate pathological changes, they may increase susceptibility to other risk factors encountered later in life, such as alcohol consumption or the use of hepatotoxic medications.

Third, the discordance between histopathological findings and evidence of genotoxicity highlights the limitations of relying solely on traditional toxicological endpoints. Therefore, regulatory evaluation of AA-containing herbs should incorporate molecular endpoints (e.g., DNA adduct measurement, mutational analysis) rather than relying exclusively on histopathological findings [17].

Fourth, from a pharmacovigilance perspective, a history of AA exposure should be considered in HCC risk assessment, particularly in Asian populations where AA-containing herbs were historically widely used [7,59]. A recent meta-analysis by Cui et al. systematically evaluated the safety outcomes associated with AA exposure, providing a scientific basis for future prevention strategies and clinical management [10].

Fifth, individuals with ARID1A deficiency may represent a particularly vulnerable population, and genetic screening for ARID1A mutations could enable personalized risk assessment for patients requiring AA-containing medications [57].

Finally, it is worth emphasizing that AA-containing herbal preparations were historically used for conditions that can now be effectively treated with safer, evidence-based pharmacotherapies. Table 2 lists historical indications of AA and their modern therapeutic equivalents. For each of these conditions, effective alternatives are available, rendering AA-containing preparations obsolete and unjustifiable in modern clinical practice.

### 7.2. Contradictory and Null Findings

It is important to acknowledge that not all studies support a causal link between AA and HCC. Several contradictory findings require careful consideration.

The AAN cohort study by Li et al. followed 337 patients with AA nephropathy for a median of 8.5 years and did not find an increased incidence of HCC. This null finding warrants critical evaluation. Possible explanations include: (1) the relatively short follow-up period given that HCC typically develops after decades of chronic liver injury; (2) a healthy worker effect, as AAN patients may have been more closely monitored and advised to avoid additional hepatotoxic exposures; (3) insufficient statistical power to detect a modest increase in HCC risk, especially if AA acts as a co-carcinogen rather than a complete carcinogen; and (4) the possibility that AA-induced hepatocarcinogenesis requires additional co-factors (e.g., viral hepatitis, metabolic dysfunction) that were less prevalent in the AAN cohort [12].

The adult mouse study by Chen et al. reported that low-dose AA exposure in adult mice did not induce long-term hepatocarcinogenic effects. This finding contrasts with studies in neonatal mice and raises the possibility of age-dependent susceptibility—the “spatiotemporal heterogeneity” hypothesis proposed by Li et al. Specifically, the developing liver may be more vulnerable to AA-induced mutagenesis than the adult liver, possibly due to higher rates of cell proliferation, differences in metabolic enzyme expression, or more efficient DNA repair in adult hepatocyte [60].

The conflicting findings across studies suggest that the carcinogenic potential of AA in the liver may be context-dependent, influenced by factors such as age at exposure, dose and duration, underlying liver disease, genetic susceptibility (e.g., ARID1A deficiency), and co-exposures. Rather than dismissing the null findings, we interpret them as important boundary conditions that help define the specific circumstances under which AA may pose a hepatocarcinogenic risk. Large-scale prospective cohort studies with long follow-up, detailed exposure assessment, and stratification by these modifying factors are urgently needed.

### 7.3. Approaches to Identify and Control Confounders in AA-Associated HCC

When evaluating suspected AA-related HCC, systematic approaches are needed to identify and control for potential confounders. Based on principles adapted from the RUCAM (Roussel Uclaf Causality Assessment Method) for herb-induced liver injury [61], we recommend the following.

When evaluating individual cases of suspected AA-related HCC, clinicians should systematically exclude a range of alternative causes. These include: viral hepatitis (e.g., hepatitis B virus [HBV] and hepatitis C virus [HCV] infection); metabolic liver diseases, including metabolic dysfunction-associated steatotic liver disease (MASLD), hemochromatosis, Wilson’s disease, and alpha-1 antitrypsin deficiency; alcohol-related liver disease, such as alcoholic liver disease or alcoholic cirrhosis; autoimmune liver diseases, including autoimmune hepatitis and primary biliary cholangitis; other drug- or herb-induced liver injuries, such as acetaminophen toxicity or other herbal hepatotoxins (e.g., pyrrolizidine alkaloids); and other HCC risk factors, including aflatoxin B1 exposure, tobacco smoking, and diabetes/obesity.

In epidemiological studies, methods to search for and control confounders include the following: First, stratification by underlying liver disease: analyses should be stratified by HBV/HCV status, MASLD, and alcohol use to determine whether AA effects are independent or synergistic with other factors. Second, adjustment for geographic and dietary co-exposures: in endemic regions where AA and aflatoxin B1 co-exposure occurs, aflatoxin B1 levels should be measured (e.g., via urinary biomarkers) and included as a covariate in the analysis. Third, use of AA-specific DNA adducts as exposure biomarkers: unlike self-reported herbal use, which is subject to recall bias, objective measurement of AA-DNA adducts in biospecimens can provide quantitative exposure assessment. Fourth, propensity score matching: this can be used in observational studies to balance known confounders between AA-exposed and unexposed groups. Fifth, sensitivity analyses: for example, restricting the analysis to individuals without major confounders (e.g., HBV-negative, non-drinkers) to isolate the independent effect of AA.

Following the RUCAM principles and systematically excluding alternative causes can help more accurately assess the potential role of AA in HCC development. These conflicting findings further underscore the need for large-scale prospective cohort studies.

## 8. Conclusions and Perspectives

Based on the evidence reviewed above, we draw the following main conclusions: First, the liver is susceptible to AA-induced genotoxicity. Despite the absence of consistent histopathological lesions, AA forms persistent DNA adducts (particularly dA-AAI) in the liver and induces mutations in key cancer-related genes (Ras and p53), with mutation frequencies comparable to known hepatocarcinogens.

Second, AA exerts both genotoxic and non-genotoxic effects in the liver. The proposed dual-mechanism framework—combining direct DNA damage with inflammation-driven tumor promotion—provides a plausible hypothetical basis for AA’s potential role in hepatocarcinogenesis. Recent discoveries have added new layers of complexity, including ferroptosis inhibition, PDK4-mediated mitochondrial dysfunction, and PSMB4 as a key molecular node [48,53]. However, most of these mechanistic findings are derived from cell lines and animal models; direct validation in human HCC tissues is still lacking.

Third, genetic susceptibility plays a critical role. ARID1A deficiency dramatically enhances AA-induced liver tumorigenesis through impaired DNA repair and enhanced metabolic activation. This finding has significant implications for personalized risk assessment [57]. Although its relevance to human AA-related HCC requires further study.

Fourth, causal evidence in humans remains incomplete and controversial. Although AA-specific mutational signatures have been detected in human liver cancers and a recent meta-analysis quantified a significant association [10], large-scale prospective epidemiological studies are still needed to establish causality. Opposing evidence from long-term AAN cohort studies and adult animal models suggests that the relationship may be more complex than initially proposed [60], possibly modified by age at exposure, underlying liver disease, and genetic background.

Fifth, and most importantly from a public health perspective, prevention of AA exposure remains the single highest priority. Regulatory actions to ban AA-containing herbal products, public education about their risks, and quality control measures to prevent AA contamination of food crops are far more impactful than any mechanistic insight. Mechanistic studies should inform, not distract from, prevention efforts.

Finally, a precautionary approach is warranted. Given the widespread historical use of AA-containing herbs in Asia, the long persistence of AA-DNA adducts, and the identification of susceptible populations (e.g., ARID1A-deficient individuals), long-term use of such herbs—especially in individuals with pre-existing liver disease or genetic susceptibility—may increase HCC risk. We emphasize that this conclusion is based on cumulative mechanistic and indirect evidence; definitive proof of causation in humans remains to be established.

Looking forward, we recommend the following priorities for future research: (1) Large-scale prospective cohort studies to assess the association between AA exposure history and HCC incidence, particularly in regions with high historical use of AA-containing herbs, while accounting for age at exposure (the “spatiotemporal heterogeneity” hypothesis); (2) biomarker development utilizing ARID1A mutation status, NQO1 polymorphisms, and AA-DNA adduct levels to develop personalized risk assessment models; (3) sensitive animal models such as genetically engineered mouse models (e.g., liver-specific Arid1a knockout mice) to further validate AA as a tumor promoter or co-carcinogen; (4) mechanistic studies investigating the interplay between AA-induced DNA damage, ferroptosis inhibition, mitochondrial dysfunction, and liver inflammation, particularly the role of the IL-6/NF-κB axis and EMT in AA-exposed livers, with an emphasis on human tissue validation; (5) intervention strategies based on the discovery of oxidative stress mechanisms to evaluate the potential protective effects of antioxidants in mitigating AA-associated carcinogenesis (NQO1 inhibitors such as skullcapflavone II have shown promise in alleviating AA-induced liver and kidney injury in preclinical models); and (6) global collaboration, as AA contamination has been detected in herbal products worldwide, requires international efforts to establish regulatory standards and surveillance systems.

Finally, the question of whether AA directly causes HCC remains open and contested. Nevertheless, accumulating evidence of AA’s genotoxic and inflammatory effects in the liver, combined with the detection of AA-specific mutational signatures in human liver cancers and a recent meta-analysis quantifying a significant association, suggests that AA is a plausible risk factor for HCC. This review does not claim causality; rather, it synthesizes existing evidence to generate testable hypotheses for future research. The identification of ARID1A deficiency as a susceptibility factor and the discovery of novel mechanisms such as ferroptosis inhibition and PDK4-mediated mitochondrial dysfunction have significantly advanced our understanding. Until definitive evidence is available, a precautionary approach is justified—particularly regarding the long-term use of AA-containing herbs in patients with underlying liver disease or genetic susceptibility.

## Figures and Tables

**Table 1:** Summary of key evidence linking aristolochic acid to hepatocellular carcinoma.

Evidence Category	Model/Sample	Key Findings
Human mutational signatures	Human HCC tissues	AA-specific mutational signature (SBS22) detected in HCC tissues; 78% of Taiwanese HCC samples positive [7]
	AA-associated UTUC patients	Somatic mutation rate of 150 mutations/Mb in AA-exposed liver tissues [8]
	Human HCC tissues	First detection of AA mutational signature in human hepatic angiosarcoma [11]
	Meta-analysis	Significantly increased liver cancer risk following AA exposure [10]
Animal studies	Hupki mice	dA-AAI adducts and 650 mutated gene loci detected in mouse liver [9]
	Rodent liver	Dose-dependent H-ras mutations in rodent liver; significant elevation of H-ras codon 61 CTA mutation [40]
	Beagle dogs	Acute liver inflammation and precancerous lesions after 10-day AA administration [36]
	Adult mice	Low-dose AA exposure in adult mice did not induce long-term hepatocarcinogenic effects (controversial) [57]
Mechanistic studies	Porcine liver	AA increased hepatic synthesis of TNF-α, IL-1β, IL-6, and IL-8 [50]
	Cell and animal models	AA upregulates PDK4, promoting p53-mediated ER stress and mitochondrial dysfunction [53]
	HCC lines	AA hijacks p53 to inhibit ferroptosis via GADD45A/NRF2/SLC7A11 axis [48]
	In vitro biochemical study	AA metabolites generate free radicals that directly attack DNA [28]
	Human-derived hepatocyte mode	Functional human-induced hiHep confirmed AA-induced oxidative DNA damage [30]
Genetic susceptibility	Liver-specific Arid1a knockout mice	ARID1A deficiency accelerates AA-induced liver tumorigenesis via impaired NER repair and enhanced NQO1 expression [58]
	Human cancer databases	ARID1A is mutated in >20% of human cancers; mutations frequently occur in non-cancerous tissues of elderly individuals [58]
Controversial evidence	AAN cohort study	Long-term follow-up of 337 AAN patients did not find increased HCC incidence [12]
	Review and hypothesis	AA carcinogenic potential may be dose- and age-dependent (“spatiotemporal heterogeneity” hypothesis) [13]

**Table 2:** Historical indications of AA-containing herbal preparations and their current evidence-based pharmacotherapies.

Historical Indication	Current Evidence-Based Pharmacotherapy (Examples)
Lung heat cough, asthma	Beta-2 agonists (salbutamol), inhaled corticosteroids (budesonide), expectorants (ambroxol)
Hypertension	ACEIs/ARBs (enalapril, losartan), calcium channel blockers (nifedipine)
Rheumatoid arthritis	Methotrexate, TNF-α inhibitors (etanercept, adalimumab), JAK inhibitors (tofacitinib)
Gout	Allopurinol, febuxostat, colchicine, NSAIDs
Pain/inflammation	NSAIDs (ibuprofen, naproxen, diclofenac), acetaminophen
Skin infections/boils	Topical or systemic antibiotics (e.g., cephalexin, clindamycin, mupirocin)
Edema/“dampness”	Loop diuretics (furosemide), thiazides (hydrochlorothiazide), spironolactone
Hemorrhoids/perianal disorders	Topical anesthetics/corticosteroid ointments, phlebotonics (diosmin)

## Data Availability

Data will be made available on request.
